# Crystal structure of di-μ-hydroxido-bis­{aqua­[ethyl (1,10-phenanthrolin-3-yl)phospho­nato-κ^2^ *N*,*N*′]copper(II)} hepta­hydrate

**DOI:** 10.1107/S2056989018015724

**Published:** 2018-11-09

**Authors:** Alexander Yu. Mitrofanov, Yoann Rousselin

**Affiliations:** a Department of Chemistry, Moscow State University, Leninskie Gory, GSP-3, Moscow 119991, Russian Federation; b ICMUB, UMR CNRS 6302, Université Bourgogne Franche-Comté, 9 avenue Alain Savary, 21078 Dijon cedex, France

**Keywords:** crystal structure, ethyl 1,10-phenanthrolin-3-yl-phospho­nate, copper(II), hydrogen bonding

## Abstract

In the title compound, [Cu_2_(OH)_2_{C_12_H_7_N_2_(PO_3_C_2_H_5_)}_2_(H_2_O)_2_]·7H_2_O, two Cu^2+^ cations are bridged by two hydroxide groups, forming a centrosymmetric binuclear complex. Each Cu^2+^cation is further coordinated by the N atoms of a bidentate ethyl (1,10-phenanthrolin-3-yl)phospho­nate anion and a water mol­ecule in a square-pyramidal geometry. In the crystal, a network of O—H⋯O hydrogen bonds involving the P(O)(O^−^)(OEt) groups, bridging hydroxyl groups, coordinated and uncoordinated water mol­ecules generates a three-dimensional supra­molecular structure. The ethyl group exhibits disorder and was modelled over three sites with occupancies of 0.455, 0.384 and 0.161.

## Abstract 2

In the binuclear complex, [Cu_2_(OH)_2_(C_12_H_7_N_2_(PO_3_C_2_H_5_))_2_(H_2_O)_2_]·7H_2_O, the two Cu^2+^ cations each have a square-pyramidal geometry and are bridged by two hydroxide groups. The phenanthroline ligand in this complex acts as a counter-ion due to a negatively charged mono­ethyl­phosphoryl group. In the crystal, O—H⋯O hydrogen bonds link the cations, P(O)(O^−^)(OEt) group and water mol­ecules of crystallization into a three-dimensional supra­molecular architecture.

## Chemical context   

Although there are only a few examples reporting the synthesis of three-dimensional coordination polymers from mono­alkyl­phospho­nates in the literature, the known examples have inter­esting properties including enhanced water stability (Taylor *et al.*, 2012 ▸) and oxygen absorption (Iremonger *et al.*, 2011 ▸). Recently, we have synthesized a new class of phenanthroline ligands bearing di­eth­oxy­phosphoryl groups (Mitrofanov *et al.*, 2012 ▸) and found that they form different supra­molecular architectures, such as dimers and one-dimensional polymers with copper(II) cations, in which the metal can coordinate to both the nitro­gen atoms of the phenanthroline core and the oxygen atoms of the di­eth­oxy­phosphoryl group (Mitrofanov *et al.*, 2016 ▸). As part of a systematic study to generate stable supra­molecular architectures based on copper(II) cations and phosphoryl-1,10-phenanthrolines, we decided to investigate the use of monoesters of phosphoryl-1,10-phenanthrolines as ligands. During these studies, the title compound, which contains centrosymmetric copper(II)-based dimers and uncoordinated water mol­ecules was obtained unexpectedly.
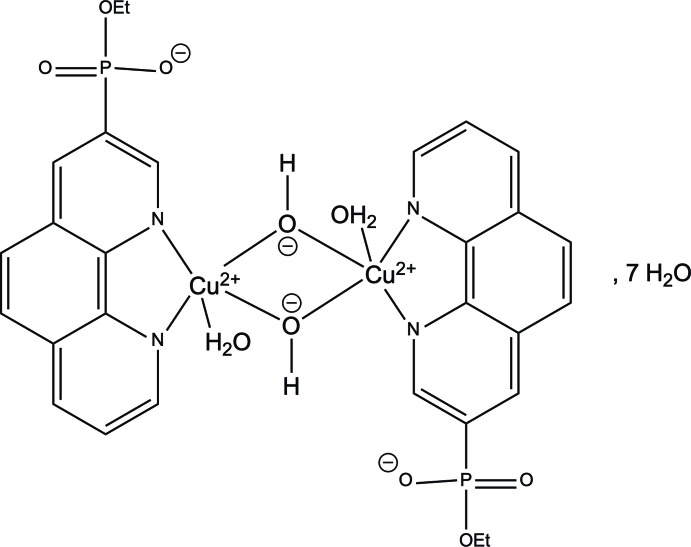


## Structural commentary   

**Figure 1:**
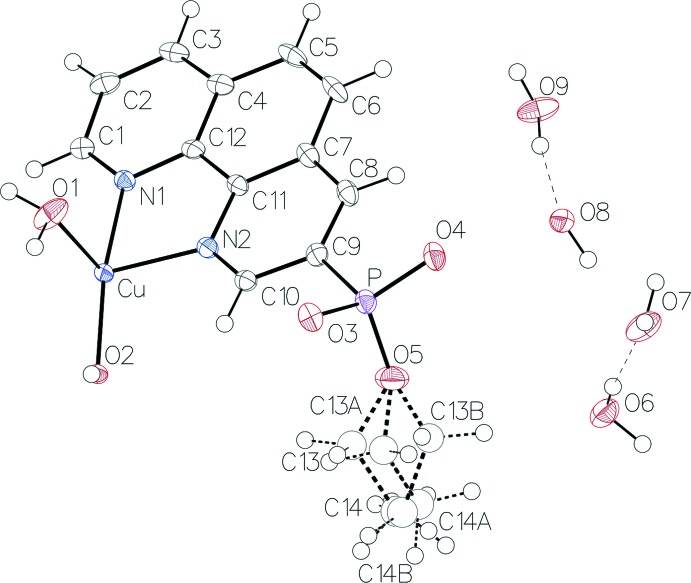
*ORTEP* view of the asymmetric unit of the title compound. Displacement ellipsoids are drawn at the 50% probability level.

**Figure 2:**
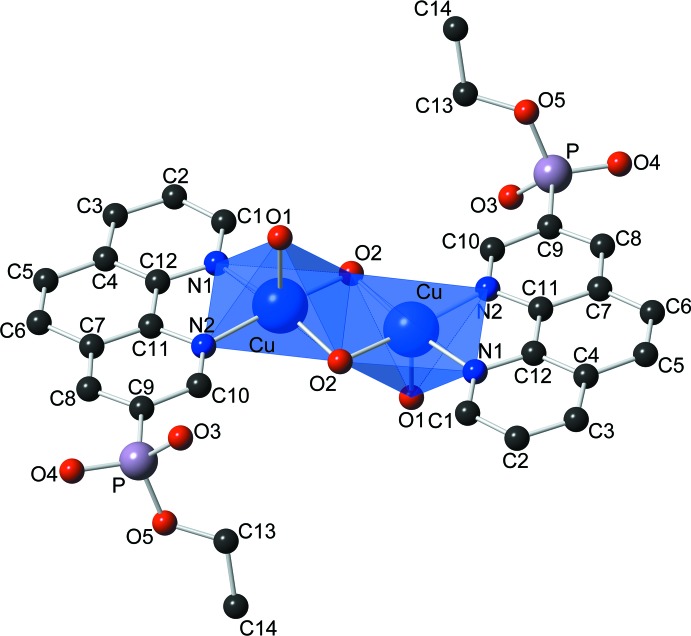
View of the title compound showing the coordination polyhedra around the copper atoms in the dimer. Only the highest occupancy components of the disordered ethyl groups (C13 and C14) are shown.

**Figure 3:**
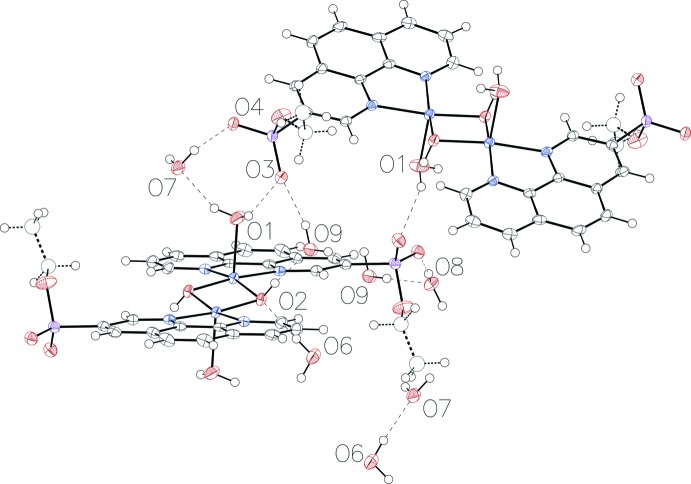
*ORTEP* view of the hydrogen-bonding inter­actions (dashed lines; see Table 1 ▸) in the title compound. Displacement ellipsoids are drawn at the 50% probability level.

The title complex crystallizes in the monoclinic crystal system in space group *C*2/c. The asymmetric unit of the compound (Fig. 1 ▸) contains one copper(II) cation, one coordinated water mol­ecule, one hydroxyl bridging group, one phenanthroline mol­ecule and 3.5 water mol­ecules. The copper(II) cation has a square-pyramidal geometry with pseudo-*C*_4v_ symmetry (Fig. 2 ▸). The spherical square-pyramidal geometry was confirmed by shape analysis using *SHAPE* software (Llunell *et al.*, 2013 ▸). The basal plane of the square-based pyramid is formed by coordination of the Cu^2+^ ion to two nitro­gen atoms of the phenanthroline ligand (N1, N2) and to the oxygen atoms of two symmetry-related hydroxyl groups (O2). The coordination of the copper atom is completed by the oxygen atom from a water mol­ecule at the apex of the square pyramid (O1). The axial Cu1—O1 distance [2.198 (2) Å] is rather longer than the equatorial Cu1—O2 bond lengths [1.948 (2) and 1.945 (2) Å], as expected from the Jahn–Teller theorem. Two of the copper centres are connected through the two bridging hydroxyl groups to form the centrosymmetric complex (Fig. 3 ▸). The pair of copper centres forms a four-cornered, planar Cu_2_O_2_ core. The two 1,10-phenanthroline mol­ecules are *trans* oriented with respect to the Cu_2_O_2_ core, forming five-membered chelate rings with the Cu atoms.

An inter­esting feature of the title complex is the short inter­metallic distance between the copper atoms in the dimer [2.8915 (9) Å]. This value is amongst the shortest Cu^II^⋯Cu^II^ distances reported in the CSD ((version 5.39, updatel May 2018; Groom *et al.*, 2016 ▸) for complexes of this type [mean value of 2.904 (13) Å for the structures reported by Zhang *et al.* (2005 ▸); Li *et al.* (2008 ▸); Lu *et al.* (2003 ▸, 2004 ▸); Arias-Zárate *et al.* (2015 ▸); Zheng *et al.* (2000*a* ▸,*b* ▸); Maldonado *et al.* (2010 ▸); Iglesias *et al.* (2003 ▸); Tu *et al.* (2009 ▸); Iqbal *et al.* (2017 ▸); Sun *et al.* (2008 ▸)].

The elongation of the apical bond length in these complexes is of comparable magnitude to that observed in the previously reported complexes. The N1—Cu—N2 angle, corresponding to the angle formed by the copper ion and the two N atoms of the 1,10-phenanthroline unit, is 82.06 (10)° for the title complex and is similar to the value for complexes of copper(II) with ligands having N and O donor atoms [mean value of 82.1 (5)° for the above-mentioned structures in the CSD].

## Supra­molecular features   

The crystal structure features a three-dimensional network of hydrogen bonds (Table 1 ▸) involving the complex mol­ecules and uncoordinated water mol­ecules (Figs. 3 ▸ and 4 ▸). Atom O1 of the coordinating water mol­ecule acts as a hydrogen-bond donor to O7 of a water mol­ecule and O3 of the phospho­nate group. The bridging hydroxide group (O2) acts as a hydrogen-bond donor to atom O9 of an uncoordinated water mol­ecule and a hydrogen-bond acceptor with water oxygen atom O6. The phospho­nate atoms O3 and O4 both form hydrogen bonds with two water mol­ecules, namely O1 and O9, and O7 and O8, respectively. The uncoordinated water mol­ecules also form hydrogen bonds with each other: oxygen atoms O6 with O7, and O9 with O8.

## Synthesis and crystallization   

The lithium salt of monoethyl 1,10-phenanthrolin-3-yl­phospho­nate was obtained from diethyl 1,10-phenanthrolin-3-yl­phospho­nate by monode­alkyl­ation with lithium bromide in 2-hexa­none at 353 K according to a literature procedure (Krawczyk, 1997 ▸). The lithium salt (29.4 mg, 0.1 mmol) was stirred with copper(I) iodide (19.1 mg, 0.1 mmol) in 1 ml of distilled water in air at room temperature. The resulting mixture was left overnight without stirring after which time, clear blue prismatic crystals were formed. The yield could not be determined because of the poor stability of the crystals out of solution.

## Refinement details   

Crystal data, data collection and structure refinement details are summarized in Table 2 ▸. The ethyl group linked to O5 exhibits disorder and was modelled over three sites with occupancies of 0.455, 0.384 and 0.161 for C13/C14, C13*A*/C14*A* and C13*B*/C14*B*, respectively. The geometric parameters of the disordered components in each group were restrained by using SADI (Sheldrick, 2015 ▸) restraints. Similar *U*_eq_ constraints were applied within the disordered parts to maintain a reasonable model with two free variable (see res file included in the CIF). Anisotropic thermal parameters were used for non-hydrogen atoms, except for the disordered ethyl group.

All C-bound H atoms were placed at calculated positions [C—H = 0.95 Å (aromatic), C—H = 0.98 Å (meth­yl), and C—H = 0.99 Å (methyl­ene)] and refined using a riding model with *U*_iso_(H) = 1.2*U*_eq_(CH), 1.5*U*_eq_(CH_3_) or 1.2*U*_eq_(CH_2_). All water mol­ecules were included as rigid groups (H—O—H 104.5° and O—H 0.87 Å). The lattice water mol­ecules were allowed to refine using AFIX 6 refinement (rotation around the O pivot atom and riding of the H atoms on the O atom for translations) whereas the refinement of coordinating water mol­ecules were handled by AFIX 7 (perpendicular rotation of the group around the Cu—O axis).

## Supplementary Material

Crystal structure: contains datablock(s) I. DOI: 10.1107/S2056989018015724/cq2028sup1.cif

Structure factors: contains datablock(s) I. DOI: 10.1107/S2056989018015724/cq2028Isup2.hkl

Supporting information file. DOI: 10.1107/S2056989018015724/cq2028Isup3.cdx

CCDC reference: 1877346

Additional supporting information:  crystallographic information; 3D view; checkCIF report

## Figures and Tables

**Figure 4:**
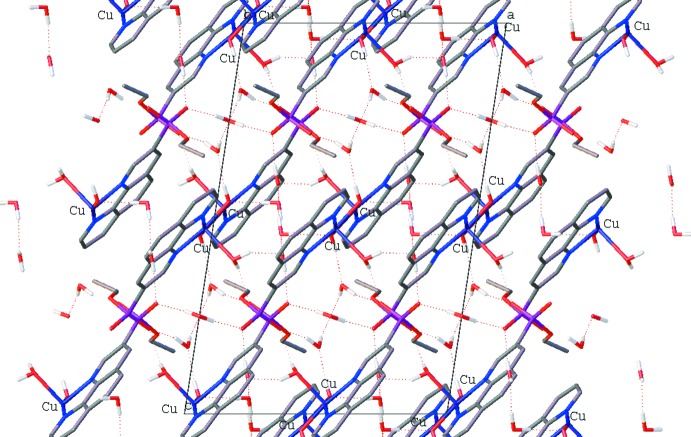
View of the hydrogen-bonded network along the *b* axis.

**Table 1:** Hydrogen-bond geometry (Å, °)

*D*—H⋯*A*	*D*—H	H⋯*A*	*D*⋯*A*	*D*—H⋯*A*
O1—H1*A*⋯O3^i^	0.89	2.04	2.759 (3)	138
O1—H1*B*⋯O7^ii^	0.89	1.91	2.733 (3)	154
O8—H8*A*⋯O4^iii^	0.86 (2)	1.85 (2)	2.709 (3)	175 (4)
O7—H7*A*⋯O4^iii^	0.87	1.85	2.697 (3)	166
O7—H7*B*⋯O6^iv^	0.87	1.91	2.731 (4)	157
O6—H6*A*⋯O2^v^	0.87	1.88	2.747 (3)	177
O6—H6*B*⋯O7	0.87	1.95	2.812 (4)	173
O9—H9*A*⋯O8	0.87	1.93	2.793 (4)	171
O9—H9*B*⋯O3^vi^	0.87	1.89	2.736 (3)	164
O2—H2⋯O9^vii^	0.84 (2)	1.89 (2)	2.727 (3)	173 (4)

Symmetry codes: (i) 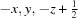; (ii) 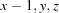; (iii) 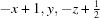; (iv) 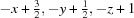; (v) 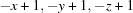; (vi) 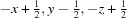; (vii) 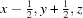.

**Table 2:** Experimental details

Crystal data	
Chemical formula	[Cu_2_(OH)_2_(C_14_H_12_N_2_PO_3_)_2_(H_2_O)_2_]·7H_2_O
*M*_r_	897.69
Crystal system, space group	Monoclinic, *C*2/*c*
Temperature (K)	115
*a*, *b*, *c* (Å)	13.3883 (4), 14.0448 (4), 20.1547 (5)
β (°)	98.702 (2)
*V* (Å^3^)	3746.18 (18)
*Z*	4
Radiation type	Cu *K*α
μ (mm^−1^)	2.89
Crystal size (mm)	0.09 × 0.09 × 0.05

Data collection	
Diffractometer	Bruker D8 VENTURE
Absorption correction	Numerical (*SADABS*; Bruker, 2012 ▸)
*T*_min_, *T*_max_	0.866, 0.949
No. of measured, independent and observed [*I* > 2σ(*I*)] reflections	25890, 3420, 2681
*R*_int_	0.073
(sin θ/λ)_max_ (Å^−1^)	0.604

Refinement	
*R*[*F* ^2^ > 2σ(*F* ^2^)], *wR*(*F* ^2^), *S*	0.041, 0.102, 1.03
No. of reflections	3420
No. of parameters	261
No. of restraints	8
H-atom treatment	H-atom parameters constrained
Δρ_max_, Δρ_min_ (e Å^−3^)	0.44, −0.38

Computer programs: *APEX3* and *SAINT* (Bruker, 2013 ▸), *SUPERFLIP* (Palatinus & Chapuis, 2007 ▸; Palatinus & van der Lee, 2008 ▸; Palatinus *et al.*, 2012 ▸), *SHELXL* (Sheldrick, 2015 ▸) and *OLEX2* (Dolomanov *et al.*, 2009 ▸).

## supplementary crystallographic information

### Crystal data

**Table:**

[Cu_2_(OH)_2_(C_14_H_12_N_2_PO_3_)_2_(H_2_O)_2_]·7H_2_O	*F*(000) = 1856
*M**_r_* = 897.69	*D*_x_ = 1.592 Mg m^−^^3^
Monoclinic, *C*2/*c*	Cu *K*α radiation, λ = 1.54178 Å
*a* = 13.3883 (4) Å	Cell parameters from 8438 reflections
*b* = 14.0448 (4) Å	θ = 4.4–68.5°
*c* = 20.1547 (5) Å	µ = 2.89 mm^−^^1^
β = 98.702 (2)°	*T* = 115 K
*V* = 3746.18 (18) Å^3^	Prism, clear light blue
*Z* = 4	0.09 × 0.09 × 0.05 mm

### Data collection

**Table:**

Bruker D8 VENTURE diffractometer	3420 independent reflections
Radiation source: sealed X-ray tube, high brilliance microfocus sealed tube, Cu	2681 reflections with *I* > 2σ(*I*)
QUAZAR MX multilayer optics monochromator	*R*_int_ = 0.073
Detector resolution: 1024 x 1024 pixels mm^-1^	θ_max_ = 68.6°, θ_min_ = 4.4°
φ and ω scans'	*h* = −15→16
Absorption correction: numerical (SADABS; Bruker, 2012)	*k* = −16→16
*T*_min_ = 0.866, *T*_max_ = 0.949	*l* = −24→24
25890 measured reflections	

### Refinement

**Table:**

Refinement on *F*^2^	0 constraints
Least-squares matrix: full	Primary atom site location: iterative
*R*[*F*^2^ > 2σ(*F*^2^)] = 0.041	Hydrogen site location: mixed
*wR*(*F*^2^) = 0.102	H-atom parameters constrained
*S* = 1.03	*w* = 1/[σ^2^(*F*_o_^2^) + (0.0515*P*)^2^ + 6.7842*P*] where *P* = (*F*_o_^2^ + 2*F*_c_^2^)/3
3420 reflections	(Δ/σ)_max_ = 0.001
261 parameters	Δρ_max_ = 0.44 e Å^−^^3^
8 restraints	Δρ_min_ = −0.38 e Å^−^^3^

### Special details

**Table:**

Geometry. All esds (except the esd in the dihedral angle between two l.s. planes) are estimated using the full covariance matrix. The cell esds are taken into account individually in the estimation of esds in distances, angles and torsion angles; correlations between esds in cell parameters are only used when they are defined by crystal symmetry. An approximate (isotropic) treatment of cell esds is used for estimating esds involving l.s. planes.

### Fractional atomic coordinates and isotropic or equivalent isotropic displacement parameters (Å^2^)

**Table:**

	*x*	*y*	*z*	*U*_iso_*/*U*_eq_	Occ. (<1)
Cu1	0.03103 (3)	0.41046 (3)	0.47418 (2)	0.01350 (14)	
P1	0.27188 (6)	0.43021 (6)	0.25570 (4)	0.0173 (2)	
O1	−0.09774 (17)	0.38520 (18)	0.39462 (12)	0.0323 (6)	
H1A	−0.118416	0.439542	0.374588	0.048*	
H1B	−0.150906	0.365681	0.412398	0.048*	
O2	0.04580 (15)	0.54561 (15)	0.45701 (10)	0.0154 (5)	
O5	0.35128 (17)	0.50141 (17)	0.29638 (13)	0.0345 (6)	
O3	0.18536 (16)	0.48343 (16)	0.21787 (11)	0.0228 (5)	
O4	0.32843 (15)	0.36193 (16)	0.21808 (11)	0.0220 (5)	
N1	0.04576 (18)	0.27507 (18)	0.50768 (12)	0.0146 (5)	
N2	0.12688 (18)	0.36346 (18)	0.41193 (12)	0.0150 (5)	
C1	0.0039 (2)	0.2325 (2)	0.55522 (15)	0.0188 (7)	
H1	−0.040543	0.268226	0.578156	0.023*	
C2	0.0223 (2)	0.1370 (2)	0.57317 (16)	0.0218 (7)	
H2A	−0.009216	0.108999	0.607597	0.026*	
C3	0.0863 (2)	0.0842 (2)	0.54049 (16)	0.0211 (7)	
H3	0.100262	0.019670	0.552596	0.025*	
C4	0.1310 (2)	0.1267 (2)	0.48893 (15)	0.0186 (7)	
C5	0.1968 (2)	0.0780 (2)	0.45050 (16)	0.0227 (7)	
H5	0.211978	0.012783	0.459638	0.027*	
C6	0.2380 (2)	0.1226 (2)	0.40129 (16)	0.0227 (7)	
H6	0.281681	0.088464	0.376781	0.027*	
C7	0.2161 (2)	0.2204 (2)	0.38619 (15)	0.0182 (7)	
C11	0.1522 (2)	0.2701 (2)	0.42314 (14)	0.0149 (6)	
C12	0.1089 (2)	0.2228 (2)	0.47470 (15)	0.0152 (6)	
C8	0.2541 (2)	0.2716 (2)	0.33540 (15)	0.0189 (7)	
H8	0.298779	0.241354	0.309631	0.023*	
C9	0.2272 (2)	0.3647 (2)	0.32278 (15)	0.0166 (7)	
C10	0.1627 (2)	0.4086 (2)	0.36289 (14)	0.0156 (6)	
H10	0.144260	0.473270	0.354352	0.019*	
C13	0.3287 (6)	0.5938 (5)	0.3131 (4)	0.0271 (14)*	0.455
H13A	0.283163	0.592040	0.347449	0.033*	0.455
H13B	0.292195	0.625909	0.272800	0.033*	0.455
C13A	0.3581 (7)	0.5981 (5)	0.2864 (5)	0.0271 (14)*	0.384
H13C	0.294856	0.629815	0.294241	0.033*	0.384
H13D	0.368533	0.611161	0.239685	0.033*	0.384
C13B	0.4066 (15)	0.5715 (12)	0.2717 (9)	0.0271 (14)*	0.161
H13E	0.373558	0.589254	0.226141	0.033*	0.161
H13F	0.474689	0.546434	0.267998	0.033*	0.161
C14	0.4208 (7)	0.6507 (7)	0.3394 (5)	0.0302 (17)*	0.455
H14A	0.450436	0.676894	0.301778	0.045*	0.455
H14B	0.470243	0.609523	0.366470	0.045*	0.455
H14C	0.401968	0.702908	0.367357	0.045*	0.455
C14A	0.4457 (7)	0.6355 (9)	0.3347 (6)	0.0302 (17)*	0.384
H14D	0.453452	0.703904	0.327170	0.045*	0.384
H14E	0.507569	0.602261	0.327587	0.045*	0.384
H14F	0.433339	0.624865	0.380797	0.045*	0.384
C14B	0.417 (2)	0.6584 (16)	0.3153 (13)	0.0302 (17)*	0.161
H14G	0.370025	0.707472	0.295210	0.045*	0.161
H14H	0.486549	0.682570	0.319143	0.045*	0.161
H14I	0.402066	0.642106	0.359971	0.045*	0.161
O8	0.500000	0.2589 (2)	0.250000	0.0229 (7)	
H8A	0.553 (2)	0.294 (3)	0.262 (2)	0.050*	
O7	0.72197 (18)	0.30341 (18)	0.41005 (12)	0.0306 (6)	
H7A	0.700619	0.312881	0.367616	0.046*	
H7B	0.725646	0.241773	0.414148	0.046*	
O6	0.76479 (17)	0.37808 (19)	0.54072 (12)	0.0291 (6)	
H6A	0.824029	0.403192	0.540061	0.044*	
H6B	0.746727	0.356738	0.500165	0.044*	
O9	0.47342 (19)	0.10011 (18)	0.32888 (12)	0.0319 (6)	
H9A	0.476158	0.152412	0.306016	0.048*	
H9B	0.423042	0.068669	0.306700	0.048*	
H2	0.019 (3)	0.562 (3)	0.4182 (12)	0.038*	

### Atomic displacement parameters (Å^2^)

**Table:**

	*U*^11^	*U*^22^	*U*^33^	*U*^12^	*U*^13^	*U*^23^
Cu1	0.0137 (2)	0.0102 (2)	0.0171 (2)	0.00096 (18)	0.00396 (17)	0.00079 (18)
P1	0.0160 (4)	0.0198 (5)	0.0162 (4)	0.0010 (3)	0.0029 (3)	−0.0025 (3)
O1	0.0231 (12)	0.0371 (16)	0.0335 (14)	−0.0104 (11)	−0.0058 (11)	0.0135 (12)
O2	0.0168 (11)	0.0105 (11)	0.0200 (11)	0.0005 (9)	0.0063 (9)	0.0020 (9)
O5	0.0291 (13)	0.0209 (14)	0.0499 (17)	−0.0018 (11)	−0.0057 (12)	−0.0066 (12)
O3	0.0230 (12)	0.0242 (13)	0.0215 (12)	0.0051 (10)	0.0041 (9)	−0.0003 (10)
O4	0.0191 (11)	0.0271 (14)	0.0206 (12)	0.0040 (10)	0.0052 (9)	−0.0044 (10)
N1	0.0142 (12)	0.0137 (14)	0.0154 (13)	0.0014 (10)	0.0005 (10)	−0.0011 (11)
N2	0.0135 (12)	0.0145 (14)	0.0162 (13)	0.0012 (10)	−0.0006 (10)	−0.0022 (11)
C1	0.0192 (16)	0.0160 (18)	0.0215 (17)	−0.0016 (13)	0.0039 (13)	−0.0012 (13)
C2	0.0248 (17)	0.0188 (18)	0.0204 (17)	−0.0055 (14)	−0.0009 (14)	0.0045 (14)
C3	0.0265 (17)	0.0126 (17)	0.0219 (17)	−0.0003 (14)	−0.0037 (13)	−0.0018 (14)
C4	0.0197 (16)	0.0147 (17)	0.0187 (16)	0.0009 (13)	−0.0061 (13)	−0.0009 (13)
C5	0.0279 (17)	0.0132 (18)	0.0248 (17)	0.0089 (14)	−0.0029 (14)	−0.0016 (14)
C6	0.0260 (17)	0.0193 (18)	0.0221 (17)	0.0104 (14)	0.0010 (14)	−0.0065 (14)
C7	0.0179 (15)	0.0191 (18)	0.0164 (16)	0.0049 (13)	−0.0007 (12)	−0.0039 (13)
C11	0.0136 (14)	0.0138 (17)	0.0158 (15)	0.0026 (12)	−0.0023 (12)	−0.0021 (12)
C12	0.0145 (14)	0.0135 (16)	0.0158 (15)	0.0000 (12)	−0.0041 (12)	−0.0029 (12)
C8	0.0155 (15)	0.0223 (19)	0.0178 (16)	0.0051 (13)	−0.0012 (12)	−0.0041 (13)
C9	0.0140 (15)	0.0193 (18)	0.0157 (15)	0.0007 (13)	−0.0007 (12)	−0.0022 (13)
C10	0.0158 (15)	0.0141 (17)	0.0165 (15)	0.0005 (13)	0.0008 (12)	−0.0005 (13)
O8	0.0186 (16)	0.0176 (18)	0.0324 (19)	0.000	0.0035 (14)	0.000
O7	0.0300 (13)	0.0324 (15)	0.0275 (13)	−0.0102 (12)	−0.0021 (11)	0.0124 (11)
O6	0.0241 (12)	0.0289 (15)	0.0348 (14)	−0.0055 (11)	0.0061 (11)	0.0057 (12)
O9	0.0456 (15)	0.0265 (15)	0.0209 (12)	−0.0161 (12)	−0.0041 (11)	0.0073 (11)

### Geometric parameters (Å, º)

**Table:**

Cu1—Cu1^i^	2.8915 (9)	C7—C8	1.408 (4)
Cu1—O1	2.198 (2)	C11—C12	1.428 (4)
Cu1—O2	1.945 (2)	C8—H8	0.9500
Cu1—O2^i^	1.948 (2)	C8—C9	1.369 (4)
Cu1—N1	2.018 (3)	C9—C10	1.411 (4)
Cu1—N2	2.036 (2)	C10—H10	0.9500
P1—O5	1.593 (2)	C13—H13A	0.9900
P1—O3	1.488 (2)	C13—H13B	0.9900
P1—O4	1.497 (2)	C13—C14	1.498 (8)
P1—C9	1.810 (3)	C13A—H13C	0.9900
O1—H1A	0.8878	C13A—H13D	0.9900
O1—H1B	0.8874	C13A—C14A	1.501 (8)
O2—H2	0.844 (19)	C13B—H13E	0.9900
O5—C13	1.386 (6)	C13B—H13F	0.9900
O5—C13A	1.378 (7)	C13B—C14B	1.498 (10)
O5—C13B	1.369 (9)	C14—H14A	0.9800
N1—C1	1.324 (4)	C14—H14B	0.9800
N1—C12	1.366 (4)	C14—H14C	0.9800
N2—C11	1.365 (4)	C14A—H14D	0.9800
N2—C10	1.324 (4)	C14A—H14E	0.9800
C1—H1	0.9500	C14A—H14F	0.9800
C1—C2	1.401 (5)	C14B—H14G	0.9800
C2—H2A	0.9500	C14B—H14H	0.9800
C2—C3	1.374 (5)	C14B—H14I	0.9800
C3—H3	0.9500	O8—H8A	0.861 (18)
C3—C4	1.408 (5)	O8—H8A^ii^	0.861 (18)
C4—C5	1.432 (5)	O7—H7A	0.8699
C4—C12	1.402 (4)	O7—H7B	0.8703
C5—H5	0.9500	O6—H6A	0.8699
C5—C6	1.359 (5)	O6—H6B	0.8697
C6—H6	0.9500	O9—H9A	0.8708
C6—C7	1.428 (5)	O9—H9B	0.8709
C7—C11	1.402 (4)		
			
O2^i^—Cu1—O1	97.47 (9)	C7—C11—C12	120.2 (3)
O2—Cu1—O1	96.74 (9)	N1—C12—C4	123.0 (3)
O2—Cu1—O2^i^	84.05 (9)	N1—C12—C11	117.0 (3)
O2—Cu1—N1	166.41 (9)	C4—C12—C11	120.0 (3)
O2^i^—Cu1—N1	95.49 (9)	C7—C8—H8	119.7
O2—Cu1—N2	96.68 (9)	C9—C8—C7	120.6 (3)
O2^i^—Cu1—N2	172.59 (9)	C9—C8—H8	119.7
N1—Cu1—O1	96.78 (10)	C8—C9—P1	121.1 (2)
N1—Cu1—N2	82.06 (10)	C8—C9—C10	118.5 (3)
N2—Cu1—O1	89.77 (9)	C10—C9—P1	120.4 (2)
O5—P1—C9	101.82 (14)	N2—C10—C9	122.7 (3)
O3—P1—O5	110.82 (14)	N2—C10—H10	118.6
O3—P1—O4	118.44 (13)	C9—C10—H10	118.6
O3—P1—C9	108.57 (13)	O5—C13—H13A	109.0
O4—P1—O5	108.27 (13)	O5—C13—H13B	109.0
O4—P1—C9	107.58 (14)	O5—C13—C14	112.8 (7)
Cu1—O1—H1A	110.4	H13A—C13—H13B	107.8
Cu1—O1—H1B	110.1	C14—C13—H13A	109.0
H1A—O1—H1B	103.6	C14—C13—H13B	109.0
Cu1—O2—Cu1^i^	95.95 (9)	O5—C13A—H13C	110.0
Cu1—O2—H2	113 (3)	O5—C13A—H13D	110.0
Cu1^i^—O2—H2	112 (3)	O5—C13A—C14A	108.2 (7)
C13—O5—P1	124.0 (3)	H13C—C13A—H13D	108.4
C13A—O5—P1	126.7 (4)	C14A—C13A—H13C	110.0
C13B—O5—P1	128.3 (9)	C14A—C13A—H13D	110.0
C1—N1—Cu1	129.6 (2)	O5—C13B—H13E	109.1
C1—N1—C12	118.1 (3)	O5—C13B—H13F	109.1
C12—N1—Cu1	112.35 (19)	O5—C13B—C14B	112.4 (15)
C11—N2—Cu1	111.9 (2)	H13E—C13B—H13F	107.8
C10—N2—Cu1	129.7 (2)	C14B—C13B—H13E	109.1
C10—N2—C11	118.3 (3)	C14B—C13B—H13F	109.1
N1—C1—H1	118.6	C13—C14—H14A	109.5
N1—C1—C2	122.8 (3)	C13—C14—H14B	109.5
C2—C1—H1	118.6	C13—C14—H14C	109.5
C1—C2—H2A	120.3	H14A—C14—H14B	109.5
C3—C2—C1	119.4 (3)	H14A—C14—H14C	109.5
C3—C2—H2A	120.3	H14B—C14—H14C	109.5
C2—C3—H3	120.3	C13A—C14A—H14D	109.5
C2—C3—C4	119.4 (3)	C13A—C14A—H14E	109.5
C4—C3—H3	120.3	C13A—C14A—H14F	109.5
C3—C4—C5	124.2 (3)	H14D—C14A—H14E	109.5
C12—C4—C3	117.3 (3)	H14D—C14A—H14F	109.5
C12—C4—C5	118.5 (3)	H14E—C14A—H14F	109.5
C4—C5—H5	119.1	C13B—C14B—H14G	109.5
C6—C5—C4	121.7 (3)	C13B—C14B—H14H	109.5
C6—C5—H5	119.1	C13B—C14B—H14I	109.5
C5—C6—H6	119.8	H14G—C14B—H14H	109.5
C5—C6—C7	120.4 (3)	H14G—C14B—H14I	109.5
C7—C6—H6	119.8	H14H—C14B—H14I	109.5
C11—C7—C6	119.2 (3)	H8A—O8—H8A^ii^	110 (6)
C11—C7—C8	116.7 (3)	H7A—O7—H7B	104.5
C8—C7—C6	124.2 (3)	H6A—O6—H6B	104.5
N2—C11—C7	123.1 (3)	H9A—O9—H9B	104.3
N2—C11—C12	116.7 (3)		
			
Cu1—N1—C1—C2	−179.6 (2)	C2—C3—C4—C12	1.4 (4)
Cu1—N1—C12—C4	−179.9 (2)	C3—C4—C5—C6	−179.9 (3)
Cu1—N1—C12—C11	−0.6 (3)	C3—C4—C12—N1	−1.0 (4)
Cu1—N2—C11—C7	179.1 (2)	C3—C4—C12—C11	179.7 (3)
Cu1—N2—C11—C12	0.0 (3)	C4—C5—C6—C7	−0.3 (5)
Cu1—N2—C10—C9	−178.3 (2)	C5—C4—C12—N1	178.9 (3)
P1—O5—C13—C14	−169.0 (5)	C5—C4—C12—C11	−0.4 (4)
P1—O5—C13A—C14A	−178.3 (6)	C5—C6—C7—C11	0.5 (5)
P1—O5—C13B—C14B	142.2 (16)	C5—C6—C7—C8	−178.9 (3)
P1—C9—C10—N2	179.1 (2)	C6—C7—C11—N2	−179.7 (3)
O5—P1—C9—C8	−108.2 (3)	C6—C7—C11—C12	−0.7 (4)
O5—P1—C9—C10	72.5 (3)	C6—C7—C8—C9	178.1 (3)
O3—P1—O5—C13	20.3 (5)	C7—C11—C12—N1	−178.7 (3)
O3—P1—O5—C13A	−16.6 (7)	C7—C11—C12—C4	0.6 (4)
O3—P1—O5—C13B	−61.9 (12)	C7—C8—C9—P1	−177.8 (2)
O3—P1—C9—C8	134.9 (2)	C7—C8—C9—C10	1.6 (4)
O3—P1—C9—C10	−44.5 (3)	C11—N2—C10—C9	−1.2 (4)
O4—P1—O5—C13	151.7 (5)	C11—C7—C8—C9	−1.3 (4)
O4—P1—O5—C13A	114.9 (6)	C12—N1—C1—C2	0.5 (4)
O4—P1—O5—C13B	69.5 (12)	C12—C4—C5—C6	0.2 (5)
O4—P1—C9—C8	5.5 (3)	C8—C7—C11—N2	−0.3 (4)
O4—P1—C9—C10	−173.8 (2)	C8—C7—C11—C12	178.8 (3)
N1—C1—C2—C3	0.0 (5)	C8—C9—C10—N2	−0.3 (4)
N2—C11—C12—N1	0.4 (4)	C9—P1—O5—C13	−95.1 (5)
N2—C11—C12—C4	179.7 (3)	C9—P1—O5—C13A	−131.9 (6)
C1—N1—C12—C4	0.1 (4)	C9—P1—O5—C13B	−177.3 (12)
C1—N1—C12—C11	179.3 (3)	C10—N2—C11—C7	1.5 (4)
C1—C2—C3—C4	−1.0 (4)	C10—N2—C11—C12	−177.6 (3)
C2—C3—C4—C5	−178.4 (3)		

Symmetry codes: (i) −*x*, −*y*+1, −*z*+1; (ii) −*x*+1, *y*, −*z*+1/2.

### Hydrogen-bond geometry (Å, º)

**Table:**

*D*—H···*A*	*D*—H	H···*A*	*D*···*A*	*D*—H···*A*
O1—H1*A*···O3^iii^	0.89	2.04	2.759 (3)	138
O1—H1*B*···O7^iv^	0.89	1.91	2.733 (3)	154
O8—H8*A*···O4^ii^	0.86 (2)	1.85 (2)	2.709 (3)	175 (4)
O7—H7*A*···O4^ii^	0.87	1.85	2.697 (3)	166
O7—H7*B*···O6^v^	0.87	1.91	2.731 (4)	157
O6—H6*A*···O2^vi^	0.87	1.88	2.747 (3)	177
O6—H6*B*···O7	0.87	1.95	2.812 (4)	173
O9—H9*A*···O8	0.87	1.93	2.793 (4)	171
O9—H9*B*···O3^vii^	0.87	1.89	2.736 (3)	164
O2—H2···O9^viii^	0.84 (2)	1.89 (2)	2.727 (3)	173 (4)

Symmetry codes: (ii) −*x*+1, *y*, −*z*+1/2; (iii) −*x*, *y*, −*z*+1/2; (iv) *x*−1, *y*, *z*; (v) −*x*+3/2, −*y*+1/2, −*z*+1; (vi) −*x*+1, −*y*+1, −*z*+1; (vii) −*x*+1/2, *y*−1/2, −*z*+1/2; (viii) *x*−1/2, *y*+1/2, *z*.
